# Cassava Starch–Onion Peel Powder Biocomposite Films: Functional, Mechanical, and Barrier Properties for Biodegradable Packaging

**DOI:** 10.3390/polym17192690

**Published:** 2025-10-04

**Authors:** Assala Torche, Toufik Chouana, Soufiane Bensalem, Meyada Khaled, Fares Mohammed Laid Rekbi, Elyes Kelai, Şükran Aşgın Uzun, Furkan Türker Sarıcaoğlu, Maria D’Elia, Luca Rastrelli

**Affiliations:** 1 Laboratory of Protection of Ecosystems in Arid and Semi-Arid Zones (eco-sys), Department of Biological Sciences, Faculty of Natural and Life Sciences, Kasdi Merbah University, Ouargla 30000, Algeria; chouana.toufik@univ-ouargla.dz; 2 Conservation and Valorization of Marine Resources Laboratory, National School of Marine Science and Coastal Management, Algiers 16320, Algeria; soufiane.bensalem@yahoo.fr; 3 Phoeniciculture Research Laboratory, Faculty of Natural and Life Sciences, University Ouargla, Ouargla 30000, Algeria; 4 Department of Pharmacy, Faculty of Medicine, Batna 2 University, Batna 05000, Algeria; meyada.khaled@univ-batna2.dz; 5 Scientific and Technical Research Center in Physico-Chemical Analysis (CRAPC), BouIsmaïl 42004, Algeriakelaielyes@gmail.com (E.K.); 6 Research Center in Industrial Technologies (CRTI), P.O. Box 64, Cheraga, Algiers 16014, Algeria; 7 Laboratory for Biological Oceanography and the Marine Environment (LOBEM), Faculty of Biological Sciences, Department of Ecology and Environment, University of Sciences and Technology Houari Boumediene, Algiers 16111, Algeria; 8 Department of Food Engineering, Faculty of Engineering and Natural Sciences, Bursa Technical University, 16310 Bursa, Türkiyefurkan.saricaoglu@btu.edu.tr (F.T.S.); 9 Department of Pharmacy, University of Salerno, Via Giovanni Paolo II, 132, 84084 Fisciano, Italy; mdelia@unisa.it; 10 National Biodiversity Future Center—NBFC, 90133 Palermo, Italy; 11 Dipartimento di Scienze della Terra e del Mare, University of Palermo, 90133 Palermo, Italy

**Keywords:** onion peel, starch, waste, biodegradable packaging, biodegradability

## Abstract

This study valorizes onion peel, an agro-industrial by-product rich in phenolic compounds and structural carbohydrates, for the development of cassava starch-based biodegradable films. The films were prepared using the solution casting method; a cassava starch matrix was mixed with a 2.5% glycerol solution and heated to 85 °C for 30 min. A separate solution of onion peel powder (OPP) in distilled water was prepared at 25 °C. The two solutions were then combined and stirred for an additional 2 min before 25 mL of the final mixture was cast to form the films. Onion peel powder (OPP) incorporation produced darker and more opaque films, suitable for packaging light-sensitive foods. Film thickness increased with OPP content (0.138–0.218 mm), while moisture content (19.2–32.6%) and solubility (24.0–25.2%) decreased. Conversely, water vapor permeability (WVP) significantly increased (1.69 × 10^−9^–2.77 × 10^−9^ g·m^−1^·s^−1^·Pa^−1^; *p* < 0.0001), reflecting the hydrophilic nature of OPP. Thermal analysis (TGA/DSC) indicated stability up to 245 °C, supporting applications as food coatings. Morphological analysis (SEM) revealed OPP microparticles embedded in the starch matrix, with FTIR and XRD suggesting electrostatic and hydrogen–bond interactions. Mechanically, tensile strength improved (up to 2.71 MPa) while elongation decreased (14.1%), indicating stronger but less flexible films. Biodegradability assays showed slightly reduced degradation (29.0–31.8%) compared with the control (38.4%), likely due to antimicrobial phenolics inhibiting soil microbiota. Overall, OPP and cassava starch represent low-cost, abundant raw materials for the formulation of functional biopolymer films with potential in sustainable food packaging.

## 1. Introduction

The food-processing industry generates massive amounts of solid and liquid waste during food manufacture, preparation, and consumption [1]. While such waste contributes to pollution and disposal problems, it also represents a significant opportunity: rich in carbon, biomass, and nutrients, these by-products can be transformed into renewable resources for the production of high-value materials, including for energy storage and advanced functional materials [2]. Each year, approximately 55 million metric tons of fruit and vegetable residues are produced during processing, illustrating the scale of this challenge [3]. Due to their high moisture content and microbial susceptibility, these wastes are difficult to manage. When landfilled, they decompose and release methane and other volatiles, aggravating climate change [4]. Improper disposal also causes odor, pathogen proliferation, and wildlife disturbance, with potential risks to human health. Among these residues, onion (*Allium cepa* L.) peel is of particular interest. Onion is one of the world’s most cultivated vegetables, with China leading global production (93.23 million metric tons), while Algeria ranks tenth, producing 1.76 million metric tons of onions annually [5]. Onion peels are especially rich in quercetin, containing up to 20 times more flavonoids than the edible bulb, as well as dietary fibers, fructooligosaccharides, and alkenyl cysteine sulphoxides [5,6]. Despite this richness in bioactive compounds, peels are generally discarded, since their strong aroma makes them unsuitable for animal feed or fertilizer production. Their accumulation in landfills or incineration not only entails costs but also creates additional environmental concerns. While current food packaging materials are predominantly based on vinyl polymers (PE, PVC, PVDC, etc.) that are difficult to degrade and environmentally unfriendly [7], many natural polymers offer excellent comprehensive properties, such as good biodegradability, antibacterial qualities, and mechanical strength; this remains the main research direction for future development [8]. In the development of biodegradable films, a variety of materials have been utilized, including starch, pectin, cellulose, and proteins. As a key example, starch is a highly abundant polysaccharide found in cereals, roots, and tubers, serving as a primary source of energy in the human diet [9]. Recent research advancements have focused on using biomass-derived materials, such as lignin [10] and starch reinforced with various plant-based components, including blackberry [11], rice flour [12], *Baccharis dracunculifolia* leaf [13], and date palm pits [9]. The goal of this research is to engineer multifunctional biocomposite films and coatings that combine biodegradability with enhanced mechanical, barrier, and other functional properties suitable for packaging applications. However, comprehensive studies focusing on onion peel incorporation into starch-based matrices remain limited, particularly concerning the interplay of physicochemical and mechanical properties. In this context, the present work investigates the effect of incorporating onion peel powder (OPP) into cassava starch films with the aim of providing a detailed analysis of their functional properties. This study addresses a critical knowledge gap by systematically assessing how OPP affects the mechanical, physicochemical, optical, and barrier properties of the resulting biofilms, thereby evaluating its potential as a low-cost, functional additive for sustainable food packaging.

## 2. Materials and Methods

### 2.1. OPP Preparation

The onions were purchased from a local market in the Oum El Bouaghi district of Algeria. The onion peels were washed and cut into approximately 1 × 1 cm^2^ pieces. The peels were air-dried at room temperature (around 25 °C) for 7 days under controlled conditions with ambient humidity maintained at approximately 50% and natural air flow to ensure uniform drying until reaching constant weight. The dried peels were then ground using a ball mill (Fritsch Pulverisette 7 premium line) with a grinding speed of 700 rpm for 15 min, and the resulting powder was sieved through a 45-µm pore-size sieve to obtain a uniform particle size. The onion peel powder (OPP) was hermetically sealed in airtight bottles and stored at room temperature (25 °C) in a dry environment for a maximum duration of 1 week before use to preserve its properties.

### 2.2. Film Preparation

Starch-based biocomposite films containing OPP were prepared according to the technique described by Oliveira Filho et al. [14]. The ratios between cassava starch and OPP were determined following a validated methodology, as described by Alqahtani et al., 2021 [9] who successfully investigated the effects of varying concentrations of date pit powder on a corn starch matrix. By using this established protocol, we ensured that our study of OPP incorporation into cassava starch films was scientifically sound and directly comparable to similar research in the field. This systematic approach allowed us to analyze how progressive increases in OPP content affect the film’s properties, as shown in Table 1. This optimization, designed to produce films with the desired properties, was carried out mathematically using Minitab 19 software, in accordance with the requirements of food packaging applications where high mechanical strength is essential. Subsequent validation experiments were performed, and the results presented in Table 1 show close agreement between predicted and experimental values. The optimization of the biodegradable composite composition was conducted on the basis of specific criteria and objectives for each formulation. For Formula 1 (1O), tensile strength was maximized while maintaining target values for elongation and biodegradability. Formula 2 (2O) prioritized high tensile strength and biodegradability while limiting elongation. Finally, Formula 3 (3O) was designed to optimize all three properties: tensile strength, elongation, and biodegradability (Table 2). To prepare the film, the cassava starch matrix was plasticized with a glycerol solution (2.5%) and heated at 85 °C under constant stirring during 30 min, while OPP was dispersed in distilled water at room temperature (25 °C) under constant stirring for 10 min. The OPP solution was mixed with the cassava starch solution and stirred for 2 min. 25 mL of mixture was dispersed in Petri dishes (ϕ = 10 cm) and dried for 24 h in an incubator at 25 °C.

### 2.3. Physicochemical Characterization of Films

#### 2.3.1. Biofilm Thickness

The measurement of biofilm thickness was assessed at five random locations using a digital micrometer Mahr Millitast 1082, accuracy 0.001 mm (Mahr GmbH is in Göttingen, Germany). For each formulation, the thickness measurement was recorded in triplicate.

#### 2.3.2. Determination of Moisture Content (MC)

The films ‘moisture content (MC) was determined using the method described by Carpiné et al. [15]. Pre-weighed samples were placed in aluminum capsules and oven-dried at 110 °C for 24 h. The MC was then calculated using Equation (1):

MC (%) = (M_1_ − M_2_)/M_1_ × 100 (1)

where: M_1_ and M_2_ represent the initial weight of the sample and the weight after oven drying, respectively.

#### 2.3.3. Determination of Solubility (S)

The water solubility (S) was evaluated according to Alqahtani et al. [9]. Briefly, 2 cm × 2 cm film samples were oven-dried at 105 °C for 24 h to determine their initial dry weight (M_1_). These samples were then immersed in 50 mL of distilled water in Petri dishes for 24 h. After this time, the biofilms were recovered and oven-dried again at 105 °C for 24 h to remove absorbed water, and their final dry weight (M_2_) was measured. Solubility was determined in triplicate according to Equation (2):

S (%) = (M_1_ − M_2_)/M_1_ × 100 (2)

where: M_1_ and M_2_ represent the initial and final dry weight of the film sample, respectively.

#### 2.3.4. Determination of Water Vapor Permeability (WVP)

The water vapor permeability (WVP) was determined gravimetrically according to Ilyas et al. [16] technique. Disc-shaped film samples were sealed onto centrifuge tubes containing 5× *g* of desiccant-grade silica gel. These sealed tubes were then placed in desiccators containing 200 mL of deionized water to maintain a constant relative humidity of 100% at a constant temperature of 20 °C. The weight of each film sample was monitored daily for seven consecutive days. To ensure reliable results, the WVP was determined in triplicate for each film type and expressed in units of g m^−1^ s^−1^ Pa^−1^, using the following Equation (3):

(3) $$WVP\left({gm}^{-1}s^{-1}{Pa}^{-}\right)=\frac{W\times x}{t\times A\times ∆P}$$

where: *W* is the increased weight of the centrifuge tube (g); *x* is the film thickness (m); *t* is the testing time (s); *A* is the permeation area of the film (m^2^); and Δ*P* represents the water vapor pressure difference across the film sample (2339 Pa).

#### 2.3.5. Determination of Color Parameters and Opacity

The color components of cassava starch-OPP films were analyzed using a Konica Minolta CR-10 colorimeter. The total color difference (Δ*E*) compared to a standard (L* = 97.39, a* = 0.14, b* = 1.94) was calculated using the following Equation (4):

(4) $$∆E=\frac{\left[\left(L*-L0\right)2+\left(a*-a0\right)2+\left(b*-b0\right)2\right]}{2}$$

where: (L) refers to lightness; (a) to redness-greenness; and (b) to yellowness-blueness.

The opacity of the film samples was assessed using a separate UV/visible spectrophotometer. The absorbance of each film was measured at 600 nm. Opacity corresponds to the ratio between absorbance value and film thickness [17].

### 2.4. Determination of Morphological and Structural Properties

#### 2.4.1. Scanning Electron Microscopy (SEM) and Energy Dispersive X-Ray Analysis (EDX)

Scanning electron microscopy (SEM) was used to examine the morphology and dispersion of composite components within the film structure [18]. This investigation was performed using a ZEISS EVO 15 microscope (ZEISS, Oberkochen, Germany). To enhance image quality, the samples were sputter-coated with a thin layer of gold (approx. 5–6 nm thick) to improve electrical conductivity during interaction with the electron beam. Energy-dispersive X-ray spectroscopy (EDX) analysis was carried out on the film samples to determine their elemental composition.

#### 2.4.2. Fourier-Transform Infrared Spectroscopy (FTIR) Test

Fourier-transform infrared (FTIR) spectroscopy was used to determine the functional groups present in the composite materials and to investigate potential interactions between components at the chemical level. The JASCO FTIR 4600 spectrometer (Japan Spectroscopic Co., Ltd. Tokyo, Japan) was utilized in this investigation. The technique involves directing infrared radiation through the sample and measuring the resulting spectrum [19].

#### 2.4.3. X-Ray Diffraction (XRD) Analysis

The crystalline structure and potential changes in starch-OPP films were investigated using X-ray diffraction (XRD) [20]. The analysis was performed on an ARL EQUINOX 100 diffractometer (Thermo Fisher Scientific, Waltham, MA, USA) equipped with a Cu Kα radiation source operating at 40 kV and 30 mA. Samples were scanned in the 2θ range from 10° to 30°.

#### 2.4.4. Determination of Mechanical Properties

The mechanical properties of the films, tensile strength (TS) and elongation at break (EB), were evaluated using a texture analyzer (TA. Zwick/Roell, Ulm, Germany) according to a modified method reported by Santos et al. [21]. Rectangular film strips (50 mm × 20 mm) were prepared for the tensile test. Test parameters included an initial crosshead speed of 10 mm/min and a separation distance of 50 mm. During the test, the machine software (testXpert V12.0) automatically recorded the force required to break the film (TS) and the maximum stretch before breaking (EB).

### 2.5. Determination of Thermal Decomposition Behavior

#### 2.5.1. Differential Scanning Calorimetry (DSC)

The thermal properties of the films were assessed using a differential scanning calorimetry (DSC) instrument (DSCQ20, TA Instruments, New Castle, DE, USA). To ensure accurate measurements, samples of around 10 mg were encapsulated in hermetically sealed aluminum crucibles to prevent mass loss during heating. The encapsulated samples were then subjected to a heating cycle from 20 °C to 350 °C at a constant rate of 10 °C/min under a nitrogen atmosphere with a flow rate of 50 mL/min [22].

#### 2.5.2. Thermogravimetric Analysis (TGA)

The thermal stability of the films and the influence of composite components were investigated using a thermogravimetric analysis (TGA) system NETZSCH-TG209F3, (NETZSCH-Gerätebau GmbH, Selb, Germany) [23]. Film samples weighing around 10 mg were placed in aluminum crucibles and heated in a nitrogen atmosphere (flow rate: 50 cm^3^/min) from 30 °C to 350 °C at a constant heating rate of 10 °C/min. An empty aluminum crucible was used as a reference for weight loss measurements. The TGA curves obtained were used to assess the weight loss of the films as a function of temperature.

### 2.6. Determination of Biodegradability

Biological degradation of starch-OPP films in soil was monitored over four weeks. The method was adapted from that described by Afshar and Baniasadi [24] with minor adjustments. The size sample (2 cm × 2 cm) was buried in a pot containing soil to a depth of 2 cm. The soil surface was kept moist by spraying water every 2 days. Sample weight was measured and observed after days. Equation (1) is used to calculate weight reduction according to Equation (5):

(5) $$Weight\;loss(\%)=\frac{\left(Wi-Wf\right)}{Wi}\times 100$$

where: *Wi* is the initial sample weight (g) and *Wf* is the final sample weight (g). Soil samples were collected and cleansed of any residual soil using a tissue before weight was calculated.

### 2.7. Statistical Analysis

All experiments were performed in triplicate on films prepared from two independent batches. Data are presented as the mean ± standard deviation (SD) together with 95% confidence intervals (CI). Statistical differences between groups were determined using a one-way analysis of variance (ANOVA), followed by Duncan’s multiple range test for post hoc comparisons. Effect sizes (η^2^) were also calculated to evaluate the magnitude of the observed differences. A significance level of *p* < 0.05 was used for all statistical analyses, which were conducted with SPSS software (version 22.0).

## 3. Results and Discussion

### 3.1. Films Characterization

#### 3.1.1. Film Thickness

**Figure 1:**
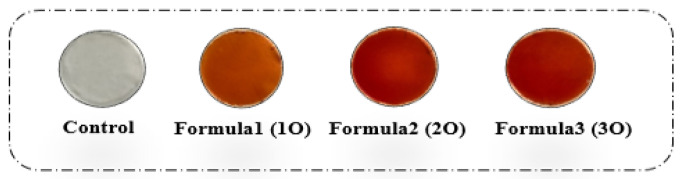
Physical appearance of cassava starch-based films containing different amounts of onion peel powder (OPP). From left to right: control (starch only), Formula 1O, Formula 2O, and Formula 3O. The image illustrates the progressive increase in opacity and color intensity with higher OPP incorporation.

The physico-chemical properties of biofilms are illustrated in Table 3. It indicates that the thickness of the films developed in this study ranged from 0.138 to 0.218 mm. Interestingly, samples containing OPP exhibited a significant (*p* ≤ 0.0001) increase in thickness compared with those made from starch. Starch films were generally the thinnest, while those with the highest OPP content were the thickest. The higher value was observed with biofilm, in which 37.68% OPP was used. Our results are in agreement with those of Mohammadi Nafchi et al. [25] and Hashemi Gahruie et al. [26] for films based on sago starch and basil seed gum. Indeed, film thickness plays a crucial role in the performance of biodegradable coatings and films. It influences various properties such as permeability, transparency, physical characteristics, optics, and mechanics [27]. The increase in film thickness observed with the addition of onion peel powder (OPP) can be attributed to a combination of two primary mechanisms. First, there is a fundamental increase in the total solid content within the film-forming solution. As the concentration of OPP increases, so does the amount of solid matter per unit area of the film, which physically results in a thicker film once the solvent has evaporated. Beyond this physical increase, a second, more complex mechanism is at play: the intermolecular interactions between the biopolymer and the additive. The bioactive compounds in OPP, particularly the phenolic hydroxyl groups, form strong hydrogen bonds with the hydroxyl groups of the cassava starch chains. This leads to a more intricate and interconnected network within the film matrix, which is not a simple physical mixture but a chemical and structural modification. The formation of this denser, cross-linked network results in a less compact arrangement of the polymer chains, ultimately increasing the overall volume and, consequently, the thickness of the film. These findings are consistent with previous studies by Capitani et al. [28] and Alqahtani et al. [9], who reported similar trends of increased thickness with higher concentrations of additives, such as Chia by-products and fruit extracts. Physical appearance of cassava starch-based films is reported in Figure 1.

#### 3.1.2. Moisture Content (MC)

The MC of control and OPP biofilms is presented in Table 3. The MC ranged from 19.159% to 32.588% for films developed from cassava starch and OPP. In particular, samples containing OPP had a lower MC than the control. Similar observations have been reported previously by Pirsa et al. [29]. This decrease in MC can be attributed to the presence of OPP, which probably prevents water molecules from penetrating the film matrix. The denser structure of OPP compared with starch may also limit the formation of interactions between water molecules and starch hydroxyl (OH) groups.

#### 3.1.3. Solubility (S)

The solubility values for cassava starch- and OPP-based films ranged from 24.039 to 25.152% (Table 3). This shows that S is comparable between OPP and control films (*p* > 0.05). However, we noted that OPP incorporation negatively influenced solubility. This result could be related to the increase in hydrophobicity and the strengthening of the polymer network in the developed films [26]. This observation may also be due to the phenolic compound content of the OPP, which can increase hydrophobicity, leading to a reduction in film solubility [30]. These interactions can probably occur between phenolic hydroxyls and carboxyl groups in the film polymers, limiting interaction with water and leading to the development of resistant [31].

#### 3.1.4. Water Vapor Permeability (WVP)

The WVP of the films, as shown in Table 3, increased significantly with increasing OPP concentration compared with the control film (*p* < 0.0001). This finding can be attributed to the hygroscopic and hydrophilic nature of onion powder. Essentially, onion powder in the films creates more sites with which water molecules can interact, facilitating their passage through the film and increasing overall WVP. Our results are quite similar to those of Azeredo et al. [32] and Otoni et al. [33] who observed a significant increase in WVP with the incorporation of hydrophilic materials. The cassava starch films with OPP, where increasing the onion peels concentration led to higher WVP. The increase observed in our study with the incorporation of OPP solution could be due to two factors. The OPP itself could introduce more hydrophilic groups into the film matrix, thus facilitating the passage of water vapor. Additionally, thicker films were formed with higher OPP content. As film thickness increases, the total distance that water vapor molecules must travel also increases, which may contribute to a slight increase in permeability. This hypothesis is supported by studies published by Sartori and Menegalli [34]. The authors demonstrated that moisture transport in films is mainly influenced by the hydrophilic portions and the overall hydrophilic/hydrophobic ratio of the components. In simpler terms, the incorporation of hydrophilic materials such as onion powder weakens the moisture barrier properties of a film. The control films, devoid of onion powder, had a denser structure due to the strong interactions between amylose and amylopectin during drying. This dense network limited the number of hydrophilic groups available to interact with water molecules. Conversely, films containing onion powder introduced more hydrophilic groups into the matrix, facilitating the diffusion of water vapor through the film and increasing its overall permeability.

The significant increase in water vapor permeability (WVP) with the incorporation of onion peel powder (OPP) is a direct result of OPP’s hygroscopic and hydrophilic nature. While this may be viewed as a drawback for packaging applications that require a moisture barrier, it is crucial to note that the primary aim of this study is to develop functional films with potential for sustainable food packaging. For future research focused on improving barrier properties, it would be relevant to explore complementary approaches. The addition of hydrophobic components to the film matrix or the application of a hydrophobic surface coating are potential strategies to reduce WVP. Furthermore, designing a multilayer packaging system, where the OPP-based film serves as the internal detection layer protected by an external layer with superior barrier properties, could offer an integrated solution to combine both functionalities. This approach would reconcile the excellent indicator function with the barrier requirements necessary for commercially viable food packaging.

#### 3.1.5. Color Parameters and Opacity

Table 4 shows the color and opacity properties of cassava starch and OPP-based films. It indicates that OPP-containing films show statistically significant differences (*p* < 0.0001) compared with the control. Furthermore, the color difference (Δ*E*) and opacity increase with the addition of OPP, resulting in films that are darker and more opaque than the control. Similar trends were reported by Nogueira et al. [11] for arrowroot starch films containing anthocyanin-rich blackberry extracts. These darker, more opaque films could be advantageous for packaging light-sensitive foods, potentially extending their shelf life by reducing light-induced degradation reactions such as lipid oxidation. In fact, OPP films exhibited lower L_0_ values (indicating in brightness), higher a_0_ values (redder, probably due to the presence of anthocyanin in OPP), and higher b_0_ values (yellower, due to the quercetin concentration). These observations are congruent with the findings of Moghadam et al. [18] for mung bean and pomegranate films which indicated that the presence of anthocyanin contributed to the redder, less glossy appearance of the films.

### 3.2. Morphological and Structural Properties

#### 3.2.1. SEM and EDX

**Figure 2:**
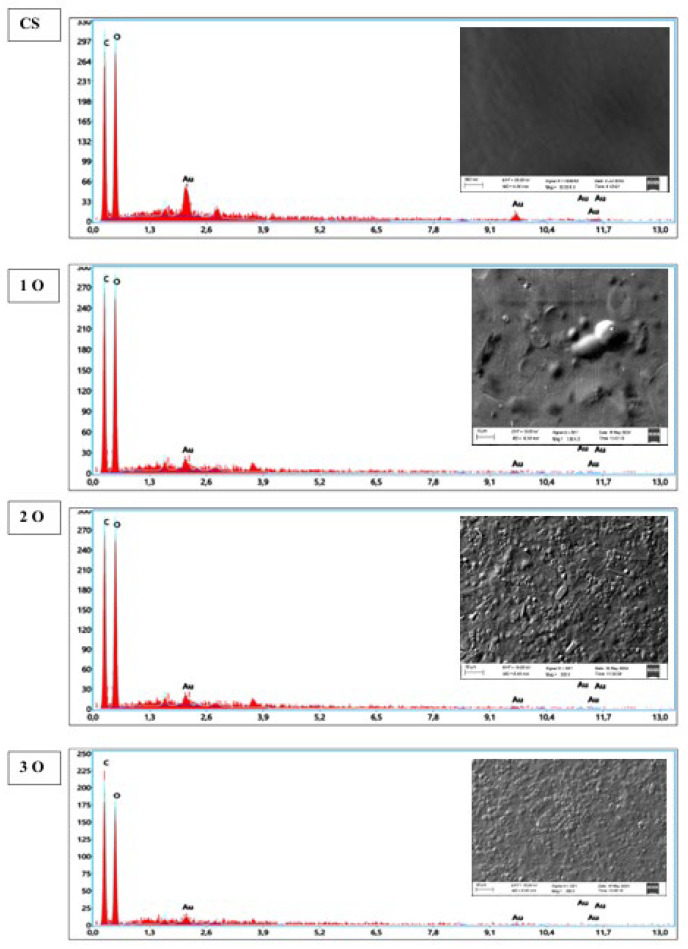
Scanning electron microscopy (SEM) micrographs and energy dispersive X-ray (EDX) analysis of cassava starch/OPP films. The control film shows a smooth and continuous surface, whereas OPP-containing films display heterogeneous distribution of microparticles (arrows). EDX spectra confirm the presence of carbon and oxygen as main elements. Magnification and scale bars are indicated.

SEM profile of OPP biofilms is illustrated in Figure 2. The OPP-free control film has a smooth, continuous surface with no cracks, breaks or openings. This continuous matrix is probably associated with the film’s elongation properties. On the other hand, SEM images of OPP-containing films revealed the presence of micron-sized OPP particles embedded in the film surface. Moreover, the distribution of these particles appears heterogeneous throughout the film. Similar results were reported by Pirsa et al. [35] for biodegradable films containing red OPP waste. The presence of particle aggregates in certain areas, as well as interstitial spaces, is a critical observation. This non-uniform dispersion could potentially lead to variations in local properties, affecting the uniformity of the mechanical and barrier characteristics across the entire film surface.

To overcome this challenge for future applications, strategies aimed at improving film homogeneity are proposed. Optimizing the film preparation conditions is a primary avenue: high-speed stirring or sonication techniques could be used to better disperse the OPP particles in the starch solution before casting. The use of dispersing agents or surfactants could also promote the integration of particles into the polymer matrix, thereby reducing agglomerates. Finally, alternative manufacturing methods, such as vacuum casting, could be investigated to minimize defects and improve film uniformity, thus ensuring more consistent and reliable properties for large-scale use. EDX analysis, shown in Figure 2, confirms the presence of carbon and oxygen as primary elements in the films. The incorporation of OPP is evident in the form of small round or oval particles in the starch film matrix. These elements presumably derive from the amylose and amylopectin components of the starch and from phenolic compounds which are abundant in onion peels. It is interesting, however, to note that these compounds contribute to the film’s strength and durability.

#### 3.2.2. FTIR

**Figure 3:**
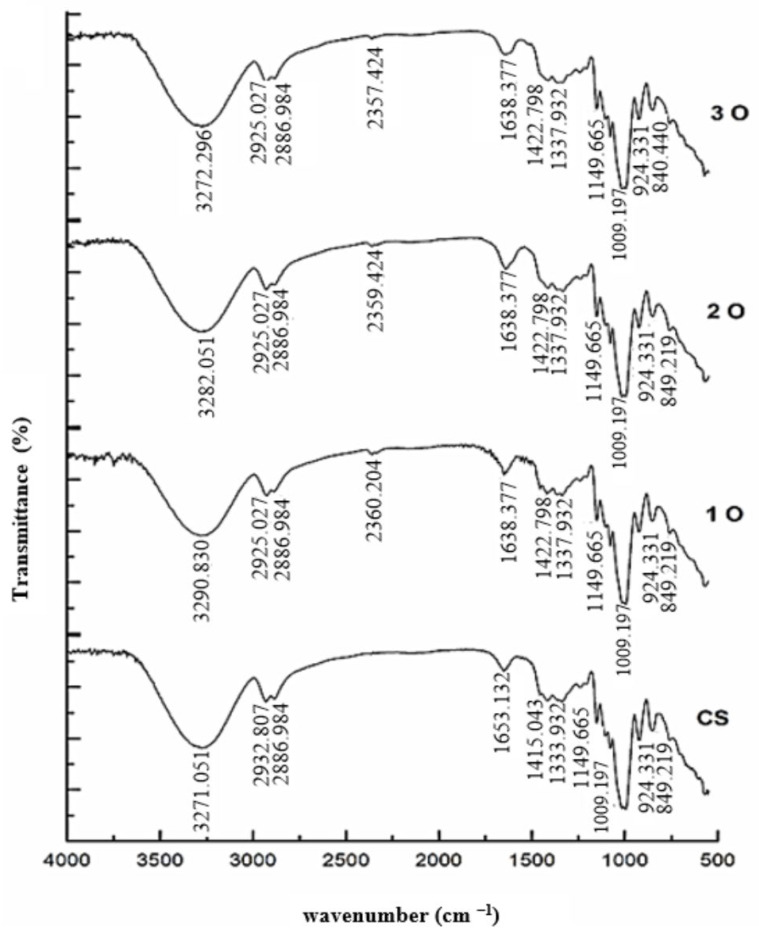
Fourier-transform infrared (FTIR) spectra of cassava starch and cassava starch/OPP films. Characteristic peaks are annotated: O–H stretching (3271 cm^−1^), C–H stretching (2932 cm^−1^), C–O and C–C vibrations (1653–1333 cm^−1^), and C–O vibrations (1150 and 989 cm^−1^). A new absorption band at 2360 cm^−1^ in OPP films is attributed to phenolic compounds. Shifts in band positions suggest electrostatic and hydrogen–bond interactions between starch and OPP.

The FTIR analysis used to investigate the interactions between the components of the starch composite films is illustrated in Figure 3. The analysis confirmed the presence of both cassava starch and OPP in the film structure by identifying their characteristic functional groups. The FTIR spectrum of the control starch film displayed a peak at 3271 cm^−1^ corresponding to O–H stretching vibrations. Also, peaks at 2932 cm^−1^, 1653 cm^−1^, 1415 cm^−1^, and 1333 cm^−1^ were attributed to stretching vibrations of –CH2– and C–H bonds, alcoholic C–O bonds, C–C bonds, and acidic C–O bonds, respectively, while peaks at 1150 cm^−1^ and 989 cm^−1^ were assigned to C–O vibrations. In the spectra of films containing OPP, a new peak appeared at 2360 cm^−1^, possibly due to stretching vibrations of the fiber and phenolic compounds present in the onion peel. Notably, compared to the control film, all peaks in composite films containing OPP were shifted to higher or lower wave numbers. Such spectral shifts are indicative of hydrogen bonding and electrostatic interactions between starch chains and phenolic hydroxyl groups of OPP. In particular, changes in the O–H stretching region (around 3271 cm^−1^) and C–O vibrations (1150–989 cm^−1^) provide direct spectroscopic evidence of hydrogen bonding within the biocomposite matrix. Comparable outcomes were also reported by Roy and Rhim [36] and Yang et al. [37] in their studies on carboxyl methylcellulose (CMC) and quercetin-modified CMC films, supporting the validity of our results.

#### 3.2.3. XRD

**Figure 4:**
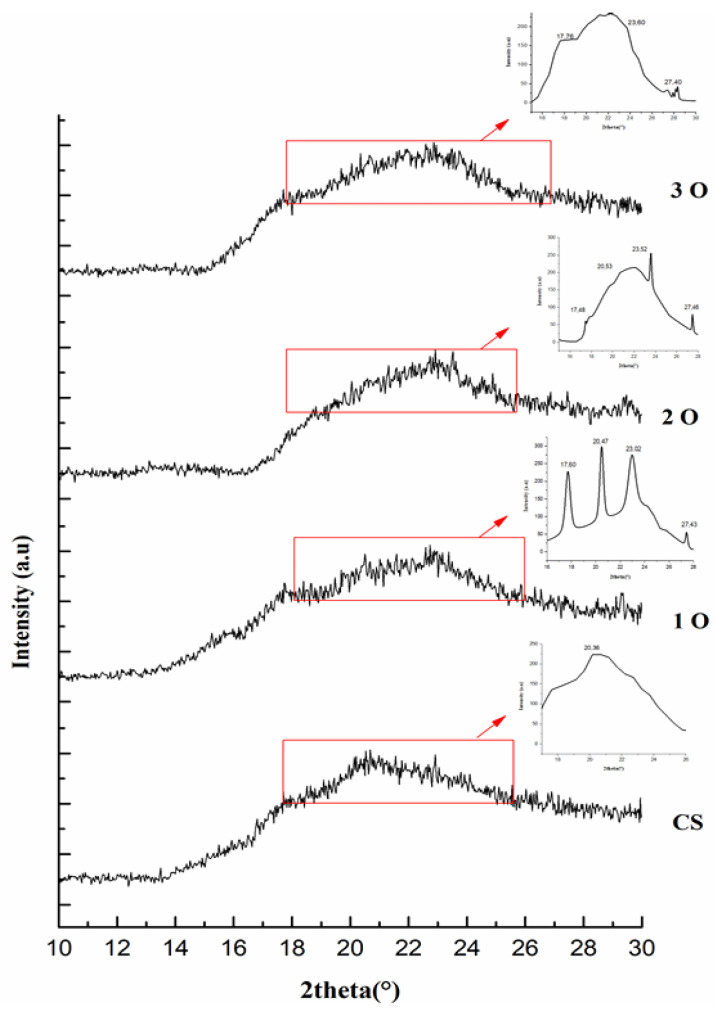
X-ray diffraction (XRD) patterns of cassava starch and cassava starch/OPP films. The control film shows a broad amorphous peak at ~20° (2θ). Additional peaks in OPP films are annotated: 10° and 27° (quercetin), 17° and 23° (cellulose). Differences in peak intensities reflect the OPP incorporation level, indicating modification of the crystalline structure.

Figure 4 displays the XRD patterns of the films examined. These patterns suggest a complex crystalline structure, potentially resembling the B-V type characteristic of tuber starches. The XRD spectrum of the control starch film showed a broad peak at around 20° (2θ), indicating an amorphous structure. In contrast, typical tuber starches show a B-type crystalline structure. The introduction of OPP is expected to disrupt the starch’s ordered crystalline structure. The presence of OPP in the films (designated 1O, 2O, 3O) is evidenced by the emergence of additional peaks in their XRD spectra compared to the control film. These new peaks, located at 2θ values of 10° and 27°, can be attributed to the presence of quercetin, a major component of onion peel [38]. In addition, the peaks at 17° and 23° likely correspond to the cellulose fibers present in OPP [39]. Moreover, the variations in peak intensity observed at 10°, 20°, and 30° may reflect the different concentrations of OPP incorporated into the starch films.

#### 3.2.4. Mechanical Properties

The mechanical properties of cassava starch/OPP films are reported in Table 5. The results revealed that OPP containing films had significant improvements in tensile strength (indicating better stress resistance) compared with the control (*p* < 0.0001). Elongation at break also showed a complex relationship with OPP concentration. While it initially increased with the addition of OPP, a further increase in OPP content led to a decrease in elongation. Formula 2O, containing 77.28% starch and 22.72% OPP, exhibited the highest tensile strength of all samples. These observations are consistent with previous research by Munir et al. [30], González et al. [40] and Alqahtani et al. [9], reporting similar trends in the mechanical properties of biodegradable films. In particular, Hun and Cennadios [41] established acceptable ranges for tensile strength (1–10 MPa) and elongation at break (10–100%) in biodegradable films. Our findings suggest that films with appropriate OPP content can achieve these benchmarks.

The enhanced tensile strength observed in OPP-containing films can be attributed to the formation of strong intermolecular hydrogen bonds. As OPP is rich in carbohydrates, including sucrose, fructose and glucose [5], it could contribute to increased tensile strength via a mechanism similar to that observed by Zhang and Han [42] with pea starch films. The influence of OPP concentration on mechanical properties observed in our study parallels trends reported for other biodegradable film systems incorporating materials such as sago starch, bovine gelatin, and pumpkin components [37,38]. Taken together, such findings imply that OPP offers a promising strategy for improving the mechanical performance of starch-based biodegradable films. The variations observed in elongation at break can be explained by the interaction between starch and OPP. At lower OPP concentrations (Formula 1O), effective interaction between the two components could facilitate efficient stress transfer throughout the film, leading to higher elongation. This is consistent with the results reported by Chaichi et al. [43] and Das et al. [44]. However, with too high an OPP content, the rigid structure of OPP could impede chain mobility in the film matrix, resulting in a decreased elongation at break. The observed decrease in elongation at break at higher OPP concentrations indicates increased brittleness of the films, which may limit their practical usability in applications requiring flexibility. While moderate OPP content provides a good balance between tensile strength and flexibility, excessive OPP incorporation results in a rigid matrix due to restricted polymer chain mobility. This trade-off suggests that optimizing OPP levels is crucial for tailoring film properties to specific needs. Additionally, future work could focus on incorporating plasticizers or blending with other biopolymers to enhance flexibility and reduce brittleness, thereby improving the overall mechanical performance and expanding the practical applications of these biodegradable films.

### 3.3. Thermal Decomposition Behavior

#### 3.3.1. DSC

**Figure 5:**
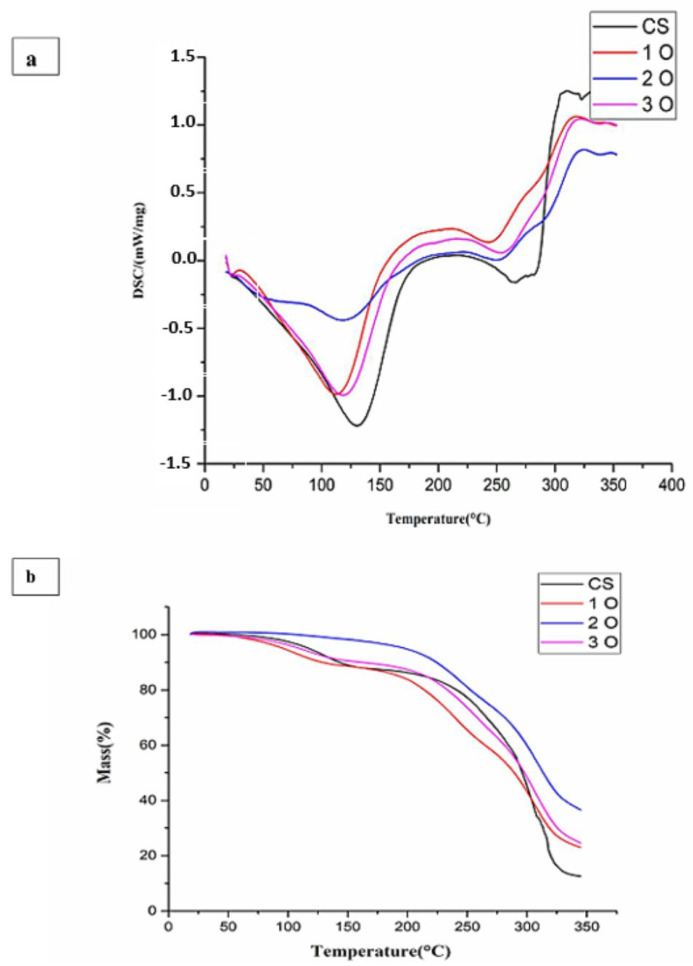
(**a**) Differential scanning calorimetry (DSC) thermograms of cassava starch and cassava starch/OPP films. Endothermic transitions are annotated: solvent evaporation/melting of retrograded crystals (70–155 °C), crystalline phase melting (~175 °C), and starch degradation (>245 °C). (**b**) Thermogravimetric analysis (TGA) curves showing three main weight-loss phases: water evaporation (<100 °C), bound water/glycerol loss (200–300 °C), and polymer chain decomposition (300–350 °C). Residual mass values are indicated, highlighting improved thermal stability in OPP-containing films.

Figure 5a presents the DSC thermograms of the starch-OPP films and the control film. All samples exhibit well-defined endothermic processes. The first peak, observed between 70 °C and 155 °C with a peak temperature (Tm) around 110–125 °C, corresponds to the evaporation of the residual solvent (probably water) used during film preparation [45]. This peak could also be attributed to the melting of crystals formed during starch retro gradation [46,47]. Interestingly, samples containing OPP displayed lower melting temperatures than the control film made from starch alone. According to the literature, certain additives or components can lower the melting temperature of thermoplastics [48,49,50]. A second broad endothermic peak, observed around 175 °C in all samples, is also associated with the melting of the starch crystalline phase [51]. Nevertheless, the maximum temperature of this process is not clearly defined. Finally, the third endothermic process involves the thermal degradation of starch (Td) [52,53]. In all cases, the films withstood temperatures of up to at least 245 °C before decomposing (Table 1). These decomposition values are consistent with those reported in the literature for highly plasticized starch films [51]. The degradation itself is most likely due to the decomposition of CH_2_OH groups in the starch [54]. Based on the thermal properties observed in the DSC analysis, starch films containing OPP appear to be suitable as coatings for various food products subjected to cooking temperatures below 245 °C.

#### 3.3.2. TGA

The TGA curves in Figure 5b show three distinctive phases of weight loss for all starch-OPP films. The initial weight loss observed below 100 °C corresponds to the evaporation of free water molecules absorbed within the film. In the second phase, between 200 °C and 300 °C, the weight loss is attributed to the loss of bound water and potentially the evaporation of glycerol, if present as a plasticizer in the film. Finally, the substantial weight loss occurring between 300 °C and 350 °C indicates that the functional groups in the polymer chains of the film have depolymerized, broke down, and decomposed. These observations are consistent with those describing thermal degradation patterns observed in chitosan/pectin-based films [55], as well as in the starch-chitosan-nanoclay bio-nanocomposite films [56]. The residual mass after thermal degradation provides insights into the thermal stability of the films. As shown in Figure 5, the control film had a residual mass of 12%, while films containing OPP displayed significantly higher values (1O: 22%, 2O: 35%, 3O: 23%). Notably, the film containing the highest OPP concentration (2O) showed the highest residual mass (35%). The above observations suggest that the incorporation of OPP enhances the thermal stability of starch films. This enhancement might be attributed to the formation of intermolecular hydrogen bonds between the functional groups present in the OPP and the starch matrix. These hydrogen bonds are likely to create a more rigid and thermally stable network within the film.

### 3.4. Biodegradability

The results of the biodegradability test are summarized in Table 6. The control film exhibited the highest biodegradability (38.38%). In contrast, films containing OPP displayed slightly lower biodegradability, ranging from 29.02% to 31.85%. Notably, formula 1O, with the lowest onion peel content, demonstrated the highest biodegradability among OPP-containing films. The incorporation of OPP appears to slightly hinder the biodegradation process. This may be attributed to the presence of phenolic compounds, which are considered potent antimicrobial agents (inhibiting the enzymatic activity of soil microbiota).

This observation highlights a crucial trade-off inherent in the design of functional and sustainable packaging materials. While a slower degradation rate may be perceived as a drawback for the product’s end-of-life, it is a direct consequence of the film’s functionality to extend food shelf-life and reduce waste, which is a major goal of sustainable packaging. Future research should strive to find an optimal balance between preservation efficiency and biodegradability. Additional studies could also evaluate the films’ degradation under industrial composting conditions, where higher temperatures and microbial activity could accelerate the decomposition process.

## 4. Conclusions

The current investigation effectively generated and characterized films based on OPP and cassava starch. OPP enrichment increased the films’ thickness and WVP while decreasing their solubility and water content. Furthermore, OPP enrichment affected the films’ color characteristics and considerably enhanced their mechanical and morphological properties. In addition, the films developed were biodegradable. It can be clearly said that OPP and cassava starch might be used as easy-to-obtain, low-cost materials in the production of biodegradable films. Although the films developed offer improved physicochemical and functional properties, further research into processing conditions and applications in the food industry is required.

## Figures and Tables

**Table 1:** Central composite design, experimental data and main results for biodegradable films made from cassava starch and onion peel powder (OPP).

Variable Code			Actual Variable		Response		
Run	X1	X2	Cassava Starch (%)	OPP (%)	Tensile Strength (MPa)	Elongation (%)	Biodegradability (%)
1	0	0	80	20	1.07 ± 0.02 ^e,f^	48.7 ± 0.02 ^f^	30.8 ± 0.05 ^e,f^
2	−1.414	0	60	20	0.75 ± 0.01 ^i^	72.1 ± 0.03 ^c^	30.0 ± 0.04 ^f^
3	1	−1	94.142	5.8579	0.58 ± 0.01 ^j^	47.6 ± 0.01 ^g^	32.1 ± 0.06 ^c^
4	1	1	94.142	34.1421	1.52 ± 0.01 ^b^	60.4 ± 0.02 ^d^	26.0 ± 0.5 ^h^
5	0	0	80	20	0.97 ± 0.02 ^g^	47.2 ± 0.05 ^h^	31.4 ± 0.03 ^c,d,e^
6	0	1.414	80	40	3.87 ± 0.01 ^a^	14.1 ± 0.03 ^l^	27.4 ± 0.02 ^g^
7	−1	1	65.858	34.1421	1.41 ± 0.02 ^c^	24.1 ± 0.05 ^k^	31.0 ± 1.14 ^d,e,f^
8	−1	−1	65.858	5.8579	0.52 ± 0.02 ^k^	73.5 ± 0.06 ^b^	36.7 ± 0.02 ^b^
9	0	0	80	20	1.08 ± 0.01 ^d,e^	47.67 ± 0.03 ^g^	30.7 ± 0.04 ^e,f^
10	1.414	0	100	20	1.11 ± 0.02 ^d^	97.1 ± 0.07 ^a^	25.2 ± 0.02 ^h^
11	0	0	80	20	1.11 ± 0.02 ^d^	52.9 ± 0.03 ^e^	32.0 ± 0.04 ^c,d^
12	0	−1.414	80	0	0.88 ± 0.02 ^h^	38.1 ± 0.04 ^j^	38.4 ± 0.2 ^a^
13	0	0	80	20	1.03 ± 0.01 ^f^	43.78 ± 0.02 ^i^	32.2 ± 0.1 ^c^
*p*-value	-	-	-	-	*p* ˂ 0.0001	*p* ˂ 0.0001	*p* ˂ 0.0001

Data are mean values ± standard deviations. Values in each column with different letters (a, b, c, d, e, f, g, h, i, j, k, l) are significantly different (*p* < 0.05).

**Table 2:** Predictive data for three biodegradable film formulations.

		Actual Variable		Response		
		Cassava Starch (%)	Onion Peel Powder (OPP) (%)	Tensile Strength (MPa)	Elongation (%)	Biodegradability (%)
1O	Prediction	72.066	21.0563	0.996	48.9	31.9
	Verification	72.066	21.0563	0.98 ± 0.02	48.7 ± 0.3	31.8 ± 0.08
2O	Prediction	77.2803	37.6878	2.75	14.1	29.1
	Verification	77.2803	37.6878	2.71 ± 0.02	14.103 ± 0.02	29.0 ± 0.02
3O	prediction	84.5616	27.7373	1.62	46.0	29.0
	Verification	84.5616	27.7373	1.62 ± 0.07	46.0 ± 0.02	29.0 ± 0.01

**Table 3:** Physicochemical properties (thickness, moisture content (MC), solubility (S), and water vapor permeability (WVP) of cassava starch/OPP films).

Films	Thickness (mm)	MC (%)	S (%)	WVP (×10^−9^ g·m^−1^·s^−1^·Pa^−1^)
Control	0.138 ± 0.04 ^c^	32.6 ± 0.3 ^a^	25.2 ± 1.9 ^a^	1.69 ± 0.02 ^c^
Formula 1 (1O)	0.176 ± 0.07 ^b^	26.2 ± 1.4 ^b^	24.0 ± 1. 6 ^a^	2.16 ± 0.23 ^b^
Formula 2 (2O)	0.218 ± 0.02 ^a^	20.5 ± 0.2 ^c^	24.0 ± 1.2 ^a^	2.77 ± 0.446 ^a^
Formula 3 (3O)	0.177 ± 0.02 ^b^	19.2 ± 0.6 ^c^	24.1 ± 1.0 ^a^	2.23 ± 0.26 ^a,b^
*p*-Value	<0.0001	<0.0001	0.57	<0.0001

Data are mean values ± standard deviations. Values in each column with different superscript letters (a, b, c) are significantly different (*p* < 0.05).

**Table 4:** Color parameters (L*, a*, b*), total color difference (ΔE), and opacity of cassava starch/OPP films.

Films	L*	a*	b*	ΔE	Opacity (nm/mm)
Control	88.9 ± 1.4	−1.07 ± 0.15	2.9 ± 0.5	8.60 ± 1.4 ^c^	5.41 ± 0.01 ^d^
Formula 1 (1O)	54.4 ± 0.9	31.6 ± 1.3	43.7± 2.4	67.7 ± 2.3 ^b^	6.08 ± 0.01 ^c^
Formula 2 (2O)	30.0 ± 1.8	37.7 ± 0.9	32. 6 ± 0.6	83.0 ± 0.9 ^a^	6.55 ± 0.01 ^a^
Formula 3 (3O)	34.8 ± 0.3	41.3 ± 1.5	33.7 ± 2.4	81.4 ± 1.8 ^a^	6.47 ± 0.44 ^b^
*p*-Value	–	–	–	<0.0001	<0.0001

Data are mean values ± standard deviations. Values in each column with different superscript letters (a, b, c, d) are significantly different (*p* < 0.05).

**Table 5:** Mechanical properties of cassava starch/OPP films.

Films	Tensile Strength (MPa)	Elongation at Break (%)
Control	0.88 ± 0.01 ^c^	38.1 ± 0.01 ^c^
Formula 1 (1O)	0.98 ± 0.02 ^c^	48.7 ± 0.3 ^a^
Formula 2 (2O)	2.71 ± 0.02 ^a^	14.1 ± 0.02 ^d^
Formula 3 (3O)	1.62 ± 0.07 ^b^	46.0 ± 0.002 ^b^
*p*-Value	<0.0001	<0.0001

Data are mean values ± standard deviations. Values in each column with different superscript letters (a, b, c, d) are significantly different (*p* < 0.05).

**Table 6:** Biodegradability of cassava starch/OPP films.

Films	Biodegradability (%)
Control	38.4 ± 0.03 ^a^
Formula 1 (1O)	31.8 ± 0.08 ^b^
Formula 2 (2O)	29.1 ± 0.02 ^c^
Formula 3 (3O)	29.1 ± 0.01 ^c^
*p*-Value	<0.0001

Data are mean values ± standard deviations. Values in each column with different superscript letters (a, b, c) are significantly different (*p* < 0.05).

## Data Availability

The original contributions presented in the study are included in the article, further inquiries can be directed to the corresponding authors.
